# Exploring barriers to human milk banking acceptability among nursing mothers in Iran using social cognitive perspectives

**DOI:** 10.1186/s13006-025-00767-9

**Published:** 2025-09-29

**Authors:** Mahnoush Karami, Zeinab Zaremohzzabieh, Mansoureh Zarean, Ali-Reza Ahmadi

**Affiliations:** 1 Faculty of Woman and Family, University of Religions and Denominations, Qom, Iran; 2 Women and Family Studies Research Center, University of Religions and Denominations, Qom, Iran; 3 Department of Social Science and Development Studies, Women Research Center, Alzahra University, Tehran, Iran; 4 Department of Biomedical Sciences, Women Research Center, Alzahra University, Tehran, Iran

**Keywords:** Breastfeeding, Human milk banking, Qualitative research, Self-efficacy, Social cognitive theory

## Abstract

**Background:**

Despite extensive global research on mothers’ intentions regarding human milk banking (HMB), its acceptability remains underexplored in non-Western contexts, particularly in Muslim-majority countries. This study investigates barriers to HMB acceptability among nursing mothers in Iran through the lens of Social Cognitive Theory (SCT), emphasizing how cultural, religious, and contextual factors intersect with maternal decision-making.

**Methods:**

A qualitative study was conducted in Tehran, Iran, between August and October 2024. Semi-structured interviews were held with twelve nursing mothers of premature infants unable to breastfeed. Data were analyzed thematically using Braun and Clarke’s six-step approach, guided by SCT to capture the interplay between personal, behavioral, and environmental influences on mothers’ decision-making regarding HMB. Rigorous strategies, including iterative coding and peer debriefing, were employed to ensure trustworthiness of the analysis.

**Results:**

Three overarching themes emerged. *Personal factors* included emotional states, risk perceptions, self-efficacy, outcome expectations, and religious beliefs and ethics. *Behavioral factors* comprised trust-based decision-making and past behavioral patterns, which shaped willingness to engage with HMB. *Environmental factors* involved institutional accessibility, social support systems, authoritative influence, and cultural norms. Findings revealed that mothers experienced emotional conflict, mistrust in milk safety, and religious concerns about milk kinship and halal practices. Institutional and logistical barriers, coupled with lack of family and community support, further reduced HMB acceptability. Nevertheless, participants emphasized that religious endorsements, transparent regulations, health professional guidance, and improved service accessibility could enhance trust and participation.

**Conclusions:**

This study highlights how reciprocal interactions among personal beliefs, behavioral patterns, and environmental contexts shape the acceptability of HMB among Iranian mothers. To improve uptake, culturally sensitive interventions are essential particularly those involving religious authorities, healthcare professionals, and awareness campaigns to address misconceptions and build trust. Strengthening institutional accessibility and transparency can further promote HMB as a viable feeding option. Future research should also examine the roles of socioeconomic status, healthcare access, and generational differences to broaden the evidence base for culturally adapted HMB policies in Muslim-majority contexts.

## Background

Exclusive breastfeeding is globally recognized as the optimal infant feeding method during the first six months of life, providing essential nutrients and critical immunological protection [1]. This practice is particularly vital for vulnerable preterm (< 37 weeks gestation) and very low birth weight (< 1500 g) infants, as human milk significantly reduces the risk of necrotizing enterocolitis (NEC) by 58–77% and late-onset sepsis by 19–42% compared to formula feeding [2, 3]. The World Health Organization (WHO) and UNICEF advocate for initiating breastfeeding within an hour of birth, exclusive breastfeeding for the first 6 months, and continued breastfeeding with complementary foods until 2 years or beyond [4]. These recommendations are echoed by other organizations like the American Academy of Pediatrics [5]. When maternal milk is unavailable, pasteurized donor human milk (DHM) from regulated milk banks serves as the recommended alternative [6]. These well-established benefits have prompted WHO and UNICEF [7] to advocate for the expansion of human milk banking (HMB) services worldwide.

Despite these advantages, milk banking remains significantly underdeveloped in non-Western regions, revealing stark disparities in implementation. While Europe currently hosts over 200 operational milk banks [8], the Middle East maintains fewer than ten facilities [9]. This imbalance persists despite comparable neonatal care needs across regions, suggesting that sociocultural factors, rather than economic or medical limitations, primarily hinder adoption. Iran exemplifies this paradox: despite high awareness of HMB benefits, participation rates remain critically low.

In response, Iran’s first human milk bank was established at Tabriz University of Medical Sciences in 2016, marking a pioneering effort to integrate modern neonatal care with Islamic principles [10]. The facility incorporates innovative, Islamic-compliant protocols, including single-donor batch processing to maintain clear lineage records, clergy-supervised consent procedures addressing milk kinship (mahram) concerns, and gender-segregated milk processing facilities [11]. Clinical outcomes demonstrate its efficacy, with recipients exhibiting 34% lower sepsis rates (*p* < .01) and 18% reduced mortality (*p* = .03) compared to formula-fed infants [11]. Yet, despite these benefits and high maternal awareness (90.6% acknowledge advantages, participation remains alarmingly low at just 12.4% [12].

Previous Iranian studies have focused on clinical outcomes and awareness levels, while intervention studies have typically employed the Theory of Planned Behavior (TPB) framework [13]. However, these approaches have proven limited, likely because they examine factors in isolation rather than their complex interactions. This gap necessitates a nuanced, qualitative exploration of the psychosocial barriers influencing maternal decision-making.

To address this, the current study adopts Bandura’s [14] Social Cognitive Theory (SCT) as its conceptual framework. SCT’s triadic reciprocal causation model conceptualizes behavior as emerging from continuous, bidirectional interactions among personal factors (e.g., religious beliefs, self-efficacy), environmental influences (e.g., cultural norms, institutional accessibility), and behavioral patterns (e.g., donation practices). This holistic approach enables an examination of how these elements interact in real-world settings, overcoming the limitations of prior quantitative studies that treated these factors separately.

Through in-depth interviews with nursing mothers, this study aims to elucidate the complex interplay of barriers limiting participation in Iran’s milk banking system. The findings seek to inform culturally sensitive, multi-level interventions that address the full spectrum of influences on maternal decision-making. By bridging medical evidence and sociocultural realities, this research contributes to global efforts to enhance neonatal health outcomes while respecting religious and cultural values in Muslim-majority contexts. Building on this aim, the study seeks to answer the following research questions grounded in SCT to explore the behavioral, personal, and environmental barriers influencing Iranian nursing mothers’ participation in HMB programs:

1. How do personal factors influence Iranian nursing mothers’ decisions to participate in HMB programs?
2. How do social support systems shape maternal attitudes and behaviors toward HMB participation?
3. What environmental factors facilitate or hinder nursing mothers’ engagement with HMB services?
4. How do behavioral factors, such as trust and modeled experiences, affect the willingness of mothers to donate or receive human milk?
5. How do nursing mothers perceive the expected outcomes of participating in HMB programs?
6. What culturally appropriate and SCT-informed strategies can be developed to improve the acceptability and utilization of HMBs in Iran?

## Literature review

### Social cognitive theory: a tool to understand HMB

**Fig. 1:**
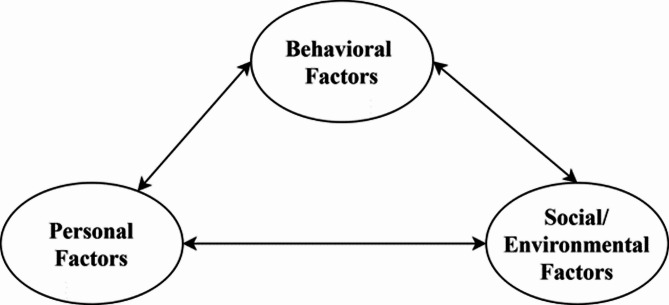
Reciprocal interactions in social cognitive theory

SCT provides a robust framework for understanding human behavior through its triadic reciprocal causation model [14]. It posits that behavior arises from continuous, dynamic interactions among three core dimensions: personal factors, environmental influences, and behavioral patterns. These factors do not operate independently; rather, they mutually shape one another in a bidirectional process termed reciprocal causation. This interaction is illustrated in Fig. 1. For example, an individual’s beliefs can shape their actions, which can alter their environment, which then influences their beliefs in turn [15]. SCT also emphasizes that individuals can control their thoughts, feelings, and behaviors, even as they are shaped by external conditions [16].

The personal factor encompasses elements like self-efficacy (an individual’s confidence in performing a task), expectations about outcomes if they act, and their emotions [17]. These internal attributes help initiate and sustain behaviors. Environmental factors include social influences—such as family opinions, cultural norms, or peer networks—and physical conditions, such as access to resources or institutional regulations. The behavioral factor pertains to individuals’ actions and how they manage their conduct over time.

These three factors facilitate understanding of complex health choices, such as donating to HMB. A mother’s decision to donate milk depends on her perceptions about sharing milk (personal), the ease of accessing a donation center (environmental), and her past experiences with donating (behavioral). These factors interact: positive experiences can enhance her confidence, while support from family can motivate her to continue donating.

SCT is particularly valuable in contexts where traditional practices intersect with modern health initiatives [18]. Unlike simpler models that focus on just one aspect of a decision, SCT considers how all these factors interrelate. This approach facilitates the development of health programs that align with a community’s culture and address multiple influences simultaneously.

### Applying SCT to HMB

This study applies SCT to explore why mothers engage with or hesitate to participate in HMB programs. Other studies have employed the TPB to examine this issue [13], but TPB focuses primarily on intended behavior and perceived control, overlooking the interaction between beliefs, surroundings, and actions. SCT addresses this gap by demonstrating how a mother’s personal convictions, social support, and access to services collectively shape her acceptance of HMB.

Research supports SCT’s applicability in understanding health decisions. For example, self-efficacy strongly predicts whether individuals adhere to exercise regimens [19] or effectively manage chronic conditions [20]. In studies on telemedicine, seeking health information (a behavior) and social support (an environmental factor) enhance online health literacy [21]. Self-efficacy and trust also shape individuals’ expectations of telemedicine adoption [22, 23].

In Iran, this study utilizes SCT to investigate the barriers preventing nursing mothers from participating in HMB programs, examining their belief systems, prior experiences, and levels of cultural and community support. This comprehensive approach offers deeper insights into maternal decision-making compared to TPB alone, informing culturally sensitive strategies to enhance HMB acceptability.

## Methods

### Research design

**Fig. 2:**
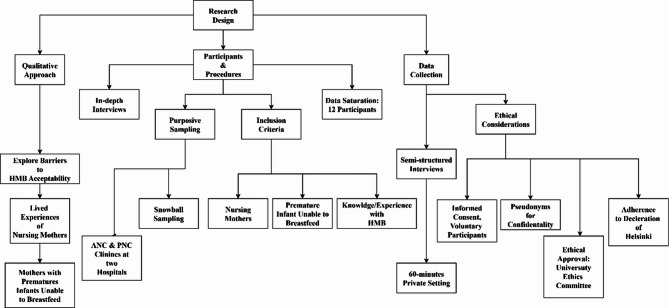
Data collection process for human milk banking study

Most prior research on HMB adoption in Iran has relied on quantitative methods [24–26]. In contrast, this study adopts a qualitative approach to explore the barriers to HMB acceptability by examining the lived experiences of nursing mothers, rather than testing predetermined hypotheses. A qualitative design was deemed most suitable [27] as it allows for a nuanced analysis of the subjective perspectives of nursing mothers with premature infants who are unable to breastfeed, as well as the specific challenges they face in accepting HMB (Fig. 2).

### Participants and procedures

The research team conducted in-depth interviews with nursing mothers of premature infants unable to breastfeed to gain a holistic understanding of their experiences and barriers to HMB participation. Participants were recruited using purposive sampling from antenatal (ANC) and postnatal care (PNC) clinics at two urban teaching hospitals affiliated with Tehran University of Medical Sciences (TUMS) and Qom University of Medical Sciences.

The first hospital, located in Tehran, is a tertiary referral center with a high-volume Neonatal Intensive Care Unit (NICU) and access to a wide population of high-risk pregnancies and premature births. The second hospital, based in Qom, also functions as a provincial referral center and has recently been involved in educational initiatives related to neonatal nutrition and maternal health. Both hospitals are situated in urban settings and serve diverse populations, including mothers from both central city areas and suburban communities.

While neither hospital currently operates a formal HMB, both have participated in pilot discussions about HMB implementation in Iran, particularly following national health initiatives under the Iranian Ministry of Health and Medical Education (MoHME) to evaluate the feasibility of milk donation programs in academic hospitals [10]. These sites were intentionally selected due to their role in regional maternal and neonatal care and their clinicians’ prior exposure to debates on HMB ethics and logistics.

To facilitate recruitment, the research team entered into formal collaboration with three neonatal nurses (two from NICU units and one from the postnatal care unit) who were briefed on the study’s objectives, inclusion criteria, and ethical protocols. These nurses, who had direct, professional access to eligible mothers as part of their clinical responsibilities, identified potential participants based on medical records and face-to-face interactions.

Importantly, nurses did not share any personal or medical information of patients with the researchers. Instead, they informed eligible mothers about the study and obtained verbal consent to refer them to the research team. This recruitment procedure is consistent with ethical standards and aligns with the Iranian National Code of Ethics in Biomedical Research [28]. Moreover, such nurse-mediated introductions are consistent with prior Iranian qualitative research in neonatal care contexts, where nurse-facilitated recruitment has been used effectively without breaching confidentiality norms [26, 29]. Snowball sampling was also employed to expand the sample beyond initial clinic-based recruitment.

The inclusion criteria were: (a) being a nursing mother, (b) having had a premature infant unable to breastfeed during the early postnatal period (e.g., due to low birth weight, weak sucking reflex, or NICU admission), and (c) possessing knowledge of or experience with HMB, either as a potential donor or recipient.

While the infants did not need to be currently in the NICU at the time of interview, most mothers had previously experienced NICU hospitalization for their infants during the critical neonatal phase, which shaped their perceptions of HMB. There was no upper age limit for the infants, as the study focused on mothers’ reflective experiences related to neonatal feeding challenges and HMB perceptions, even if their child was older than one year at the time of interview.

In line with Iranian NICU and post-NICU care studies [30], it is common for mothers to continue breastfeeding or expressing milk even after NICU discharge, especially when complementary feeding has begun but breastfeeding is still ongoing. In many cases, mothers attempted to continue lactation through pumping or wet-nursing after encountering early breastfeeding failure due to prematurity. This supports their status as ‘nursing mothers’ despite past or present breastfeeding complications.

Following qualitative research standards [31], recruitment continued until data saturation was reached—the point at which no new themes emerged from the interviews. The final sample comprised 12 participants (Table 1).

**Table 1:** Demographic profile of participants (*n* = 12)

Participant	Age	Occupation	No. Children	Age of Youngest Child
P_1_	25–30 years	Dentist	1	12–18 months
P_2_	> 30 years	Homemaker (Master’s degree)	1	12–18 months
P_3_	25–30 years	Homemaker	1	0–6 months
P_4_	25–30 years	Employee (Master’s degree)	1	12–18 months
P_5_	> 35 years	Homemaker (Bachelor’s degree)	2	0–6 months
P_6_	> 40 years	Employee (Master’s degree)	2	0–6 months
P_7_	> 30 years	Homemaker (Master’s degree)	3	0–6 months
P_8_	> 30 years	Seminary Student (Level 2)	3	12–18 months
P_9_	> 30 years	Employee (Bachelor’s degree)	1	12–18 months
P_10_	25–30 years	Student (Diploma)	1	0–6 months
P_11_	25–30 years	Homemaker (Bachelor’s degree)	2	6–12 months
P_12_	> 30 years	Employee (Master’s degree)	1	6–12 months

### Data collection

Data were collected through semi-structured, in-depth face-to-face interviews, each lasting approximately 60 min. Interviews took place in private settings and focused on participants’ experiences, perceptions, and barriers related to HMB. Prior to participation, individuals were informed of their rights, including the voluntary nature of participation and their ability to withdraw at any time. To protect confidentiality, pseudonyms were assigned to all participants. The study received ethical approval from the University Ethics Committee for Research Involving Human Subjects (IR.REC.1398.123) on 19 August 2024 and was conducted in accordance with the principles of the Declaration of Helsinki.

### Data analysis

This study adhered to the Consolidated Criteria for Reporting Qualitative Research (COREQ) checklist [32] to ensure transparency and methodological rigor throughout the research process. This included careful documentation of interviewer characteristics, data collection procedures, research team reflexivity, coding strategies, and the development of themes. The data analysis was carried out by a three-member interdisciplinary team. Two members were PhD-trained qualitative researchers with expertise in maternal and neonatal health, both of whom had previously published studies in Iranian journals focusing on breastfeeding and NICU experiences [33, 34]. The third member was a nursing faculty member with more than ten years of clinical experience in neonatal intensive care and prior training in qualitative research methods.

All three team members were female, fluent in Farsi, and familiar with the cultural context of neonatal care and breastfeeding practices in Iran. Their diverse professional backgrounds strengthened the credibility and depth of the analysis. Interview transcripts were transcribed verbatim and analyzed using MAXQDA24 software. **A** thematic analysis approach [35] was adopted, allowing inductive, data-driven coding without reliance on pre-existing frameworks.

To ensure reliability of the codes and findings, the two primary researchers independently coded the first six transcripts using line-by**-**line inductive coding. They then met to compare their coding outputs and engaged in negotiated consensus meetings to resolve discrepancies. Disagreements were discussed openly through reflexive team dialogue until consensus was achieved on how to interpret specific codes and assign them to themes. This process enhanced inter-coder reliability and minimized subjective bias. A shared coding framework (codebook) was iteratively developed from these initial transcripts. As new interviews were analyzed, the team refined the codebook in recurring meetings to incorporate novel insights and ensure alignment with participant narratives. The third team member acted as an external auditor, reviewing a random sample of coded transcripts and examining the alignment between assigned codes and emerging themes. Her feedback informed ongoing revisions to the coding structure and helped validate theme saturation.

The constant comparative method [36] was applied throughout to ensure that themes were consistent across different cases and participants. Codes were continuously compared within and across transcripts to refine categories, identify outliers, and preserve the diversity of participant experiences. Any coding discrepancies that arose beyond the initial set of transcripts were similarly addressed through team-based discussion, with all decisions grounded in participant quotations and the conceptual aims of the study. No major conflicts remained unresolved, and the team reached agreement on the final thematic structure.

Given the novelty and cultural sensitivity of HMB in Iran, an inductive approach was essential. It allowed themes to emerge organically, reflecting participants’ lived experiences without imposing theoretical constraints—an approach validated in Iranian maternal health research [37]. This rigorous, collaborative, and reflexive analytical process ensures that the findings are credible, dependable, and transferable in accordance with criteria for qualitative trustworthiness [38].

## Results

Analysis of the data revealed three main themes capturing the experiences of the study participants. These themes were classified through data analysis as: personal factors, behavioral factors, and environmental factors. Each main theme is further elaborated through the identification of sub-themes, as shown in Table 2. Personal factors were defined by five types of experiences—emotional state, risk perception, self-efficacy, outcome expectations, and religious beliefs. Behavioral factors were described as trust-based decision-making and past behavioral patterns, which affected the nursing mothers’ perceptions of accepting HMB. Finally, environmental factors captured the social aspects of the nursing mothers’ acceptance of HMB, which included institutional accessibility, social support systems, authoritative influence, and cultural norms. Each theme is discussed in detail below.

**Table 2:** Summary of themes

Theme	Sub-themes	*n*
Personal factors	Emotional state	10
	Risk perception	9
	Self-efficacy	7
	Outcome expectations	6
	Religious beliefs and ethics	9
Behavioral factors	Trust-based decision making	8
	Past behavioral patterns	7
Environmental factors	Institutional accessibility	8
	Social support system	7
	Authority influence	6
	Cultural norms	6

## Emerged themes

### Personal factors

The findings revealed that personal factors played a pivotal role in shaping mothers’ attitudes toward HMB, intertwining emotional, practical, and ethical considerations that influenced their perceptions.

#### Emotional state

Emotional factors, particularly feelings of guilt and maternal identity, emerged as strong determinants of mothers’ attitudes. Many participants experienced profound shame and a sense of failure when unable to breastfeed. This distress was exacerbated by societal and familial expectations, which framed breastfeeding as essential to motherhood. Mothers reported experiencing anxiety, frustration, and, in some cases, depressive symptoms due to their inability to provide breast milk directly. P_11_ stated:

*“Shame on me if I can’t breastfeed; I feel like a bad mom. Breastfeeding is not just about feeding—it’s about bonding*,* nurturing*,* and fulfilling my role as a mother. I feel pressure from society*,* family*,* and even myself to do it naturally. If I rely on HMB*,* I worry that I’m failing my child or that others will judge me for not being ‘enough’ as a mother.”*

This sentiment illustrates how societal and familial pressures intensified mothers’ emotional burdens, heightening fears of inadequacy associated with using HMB. Participants described a conflict between their desire to provide optimal nutrition and the emotional toll of their perceived failure.

#### Risk perception

Concerns regarding hygiene, contamination, and the risk of disease transmission emerged as prominent barriers to the acceptability of human milk banks. Participants consistently expressed skepticism about the screening processes for donor mothers, particularly in relation to their personal health, hygiene practices, and potential substance use. These concerns highlighted a broader lack of trust in the milk banking system, which many participants perceived as lacking transparency and adequate regulation.

As one participant (Participant 8) explained:

*“What if it’s contaminated? I worry about diseases. I don’t know how the milk was collected*,* stored*,* or handled. Was it kept in a clean and safe environment? Could it carry infections or bacteria that might harm my baby? Without strict regulations and proper screening*,* I’d always be anxious about the risks.”*

This statement reflects the widespread mistrust rooted in uncertainty surrounding quality control measures—such as microbiological testing, storage standards, and donor lifestyle evaluations. Participants emphasized that a lack of awareness and assurance about these procedures contributed to heightened fears about possible contamination or infection, ultimately discouraging the acceptance of donor milk.

To address these concerns, many mothers emphasized the need for transparent and accessible information about human milk bank operations. They recommended the implementation of rigorous screening protocols to detect alcohol and drug use, as well as infectious diseases among donor mothers. Additionally, they called for the public dissemination of clear hygiene standards related to the collection, handling, and storage of human milk. Finally, participants highlighted the importance of oversight by credible health authorities and/or respected religious leaders to enhance public trust in the system and ensure cultural and ethical alignment.

#### Outcome expectations

Participants held mixed views regarding the long-term efficacy of banked milk. While many acknowledged breast milk’s superiorities over formula in terms of nutrition, some questioned whether stored milk retained the same immunological benefits as fresh milk. P_6_ articulated this ambivalence:

*“It’s better than formula for the first month*,* but not after. Breast milk is important for immunity and nutrition in the early days*,* but after that*,* I feel like formula or regular food is just as good. Plus*,* I don’t know if stored or banked milk keeps the same benefits as direct breastfeeding.”*

This statement reflects an initial recognition of breast milk’s benefits but also expresses doubts about its sustained efficacy compared to other feeding alternatives. Participants emphasized the need for more scientific evidence on the long-term benefits of stored human milk.

#### Self-efficacy

Self-efficacy strongly influenced mothers’ willingness to participate in HMB, with some expressing openness under the right conditions. Many participants stated that they would consider donating or receiving milk if the process was accessible, straightforward, and well-regulated. P_4_ explained:

*“I’d contribute if it’s easy and I know it helps someone. If the process was simple*,* safe*,* and I was sure my milk was going to a baby in need*,* I’d be happy to help. But if it’s complicated*,* requires travel*,* or has too many restrictions*,* I wouldn’t bother.”*

This response indicates that confidence in HMB depended on a streamlined and transparent process, while logistical barriers significantly reduced enthusiasm. Participants also highlighted the need for educational campaigns to address misconceptions and improve awareness about milk banking.

#### Religious beliefs and ethics

Religious and ethical considerations added layers of complexity, profoundly shaping attitudes toward both donating and receiving milk. While religious beliefs—particularly those related to milk kinship, lineage, and halal practices—formed the core of participants’ concerns, ethical dimensions such as transparency, informed consent, and moral responsibility also emerged as critical. For many mothers, decisions were not solely based on what is religiously permissible, but also on what they perceived to be morally right for their child, family, and community.

Some mothers hesitated due to uncertainties about the donor’s lifestyle (e.g., whether the donor smoked, used medication or alcohol, or adhered to religious dietary practices), dietary habits, and potential religious implications, such as whether the donor was in a state of ritual purity (ṭahārah) when expressing the milk. P_3_ stated:

*“I don’t know who the donor is*,* what they eat*,* if it’s halal or haram. I worry about their lifestyle*,* their health conditions*,* and whether they follow religious dietary and ethical guidelines. What if they consume something forbidden in my faith? What if they have illnesses that could affect the milk? These uncertainties make it difficult for me to trust the process completely.”*

Her concerns underscore how religious and health-related uncertainties undermined trust in the system. Similarly, P_1_ raised concerns about milk kinship, stating:

*“Milk makes siblings; I don’t want my child tied to a stranger. What if*,* in the future*,* my child unknowingly meets and forms a relationship with someone who is their milk sibling? Without clear records or transparency*,* how can we ensure that such relationships don’t happen? It worries me because*,* in our faith*,* milk kinship is just as significant as blood ties.”*

This statement highlights the cultural and religious significance of milk kinship, emphasizing the need for clear documentation and transparency to address these concerns.

### Behavioral factors

Behavioral factors played a significant role in shaping mothers’ attitudes toward HMB, particularly regarding institutional trust and past experiences with milk-sharing.

#### Trust-based decision-making

Trust in HMB institutions emerged as a major concern, with participants expressing hesitation about using milk from anonymous donors. Many mothers preferred milk from known and trusted sources rather than relying on formal milk banks. P_7_ explained:

*“If I knew the donor was a good*,* religious person*,* I’d use it. I need to be sure that they follow halal practices*,* have good moral character*,* and live a healthy lifestyle. It would also be important to have a clear record of the donation so my child knows their milk siblings in the future. Trust and transparency matter a lot when it comes to something as personal as feeding my child.”*

Some participants also considered the gender of the donor’s child as a critical factor. P_3_ noted:

If the donor’s child is the same gender as mine, I’d be even more careful,” one mother noted. “Boys or girls of the same age might grow close later in life, so I need to make sure we know exactly who their milk siblings are to prevent any future issues. It’s not just about health — it’s about protecting them socially and religiously too.

This illustrates how religious and social norms—especially concerns about future interactions between milk siblings of the same gender—shape maternal trust in HMB systems. Participants emphasized the need for transparent documentation and detailed donor records to ensure compatibility and avoid potential religious or ethical conflicts.

#### Past behavioral patterns

Participants’ past experiences with breastfeeding and informal milk-sharing also influenced their perspectives. Many mothers who had previously engaged in community-based milk-sharing viewed HMB with skepticism, believing that a personal connection with the donor was essential for trust. P_10_ shared:

*“I’d rather give to a neighbor than a hospital bank. If I know the person*,* I can trust their lifestyle*,* their hygiene*,* and their religious practices. It feels more natural to share within my community than to take milk from an anonymous source.”*

This preference for local, informal sharing reflects a broader mistrust of institutionalized milk banking systems. It underscores the importance of social connections and community-based solutions in shaping mothers’ choices.

### Environmental factors

Environmental factors, including **l**ogistical accessibility, social support, authoritative influence, and cultural norms, played a crucial role in shaping mothers’ attitudes toward HMB.

#### Institutional accessibility

Practical challenges related to accessibility emerged as a significant barrier. Many participants found the process of obtaining milk from formal milk banks inconvenient and burdensome. P_5_ articulated this difficulty:

*“It’s hard to go to a bank every day; I’d rather use formula if it’s easier. With a newborn*,* I already have so much to manage*,* and making regular trips to a milk bank feels overwhelming. If there were a more convenient system—maybe home delivery or local collection points—I might consider it. But right now*,* formula is just more accessible and practical.”*

This sentiment underscores the need for more flexible and accessible distribution systems to make HMB a viable option for mothers facing logistical constraints. Some participants suggested alternative models, such as mobile milk banks or integrating milk donation into existing healthcare facilities, to ease access and improve participation rates.

#### Social support systems

Family and social influences played a pivotal role in shaping mothers’ decision-making, with relatives often exerting strong influence over their choices. P_9_ described the weight of familial opinions:

*“My mom says it’s not clean*,* my husband decides what’s best. In our family*,* elders have a big say in how we raise our children*,* and if they don’t trust milk banks*,* it’s hard for me to go against them.”*

This highlights the hierarchical nature of family decision-making, where societal and familial pressures can limit a mother’s autonomy. Many mothers expressed concerns about potential conflicts or judgment if they chose to use HMB against their family’s wishes.

#### Authoritative influence

Endorsement from trusted authorities, particularly healthcare professionals and religious scholars, significantly influenced attitudes toward HMB. P_2_ expressed a need for professional reassurance:

*“If a doctor said it’s safe and good*,* I’d trust it more. If a paediatrician or religious scholar endorsed it and explained the safety measures*,* I’d feel more comfortable.”*

This suggests that institutional trust could be strengthened through educational initiatives led by credible experts. Public awareness campaigns, featuring testimonials from respected medical professionals and religious leaders, could help alleviate concerns and improve acceptance rates.

#### Cultural norms

Many participants noted that HMB is not widely accepted in their social and religious contexts. P_12_ observed:

*“In Europe*,* it’s common*,* but here people don’t accept it easily. Without cultural acceptance and religious approval*,* it’s hard for people to trust it.”*

This underscores the importance of cultural adaptation and religious endorsement in fostering acceptance of HMB. Addressing cultural concerns through religious discourse, community discussions, and awareness programs may facilitate greater acceptance of HMB within conservative societies.

## Discussion

This study, grounded in SCT, examined the interplay of personal, behavioral, and environmental factors in shaping barriers to the acceptability of HMB among nursing mothers in Iran. SCT posits that human behavior is shaped by a dynamic, reciprocal interaction among cognitive, affective, and environmental influences rather than being determined solely by internal dispositions or external forces. Our findings highlight how maternal emotional states, risk perceptions, self-efficacy, outcome expectations, and religious beliefs shape individual attitudes toward HMB. Furthermore, trust-based decision-making and past behavioral patterns significantly influenced mothers’ acceptance or resistance to HMB. Environmental factors, including institutional accessibility, social support systems, authoritative influence, and cultural norms, emerged as critical determinants of HMB perceptions and decision-making processes. These findings align with Bandura’s [14] triadic reciprocal causation model, which illustrates how individual, social, and contextual elements collectively shape maternal behaviors related to milk banking.

The results demonstrated the emotional state of recipient mothers as a prominent subtheme within personal factors, with guilt and maternal identity frequently reported. Consistent with Mondkar et al. [39], many mothers experience guilt due to limited access to HMB, yet witnessing their infants thrive on donated milk often fosters acceptance and emotional relief. Similarly, Mackenzie et al. [40] found that while recipient mothers may initially feel vulnerable or inadequate, access to HMB positively influences their mental well-being.

Risk perception also emerged as a major concern, aligning with prior research that highlights bacterial contamination, potential substance adulteration, and donor health conditions (e.g., drug use) as primary risks in informal milk sharing [41–43]. The current study observed a low prevalence of formal milk-sharing agreements, indicating that milk sharing often occurs informally, with recipients accepting inherent risks. Recipient mothers frequently inquired about donors’ health screening, medical history, pregnancy circumstances, substance use, and hygiene practices during milk expression. Although a U.S.-based study of 867 women found that 9.3% of recipients did not require donor screening [44], the Academy of Breastfeeding Medicine’s protocol recommends a thorough evaluation of donors’ medical and social histories [45].

The emotional experiences of recipient mothers further intersect with self-efficacy and outcome expectations, particularly regarding HMB accessibility. Consistent with Bandura’s [46] concept of “enactive attainment,” nursing mothers’ self-efficacy improves when they successfully navigate breastfeeding and milk donation. However, when access to HMB is limited, mothers may experience heightened guilt or inadequacy. Similar to midwives’ satisfaction with successful breastfeeding support [47], recipient mothers experience relief and increased self-efficacy when their infants benefit from donated milk. Additionally, perceived health benefits of donor milk, akin to positive breastfeeding outcome expectations, enhance maternal acceptance of HMB. These findings suggest that emotional responses, such as guilt, when coupled with successful experiences of milk donation, can improve self-efficacy and shape positive perceptions of HMB. This is consistent with research linking self-efficacy to milk donation intentions [48].

However, religious beliefs remain a significant barrier to HMB acceptance, particularly in Islamic contexts where milk donation is believed to establish kinship ties that could create marriage restrictions [49, 50]. This religious concern complicates the establishment of HMB in Muslim countries, with some mothers refusing to donate or accept milk due to fears of violating kinship norms [51]. Al-Naqeeb et al. [52] advocate for culturally sensitive approaches, suggesting that HMBs in Muslim-majority countries should implement a mutual identification system between donors and recipients to address these concerns.

Another key theme, behavioral factors, consists of two subthemes: trust-based decision-making and past behavioral patterns. These barriers significantly influence HMB accessibility. Trust in healthcare professionals plays a crucial role in shaping mothers’ decisions to accept HMB. Prior studies indicate that mothers feel reassured by physicians’ recommendations and medical expertise [39, 47]. In formal HMB settings, trust in the screening and pasteurization process enhances confidence in donor milk safety. In contrast, in informal milk sharing, trust is primarily built through direct communication and personal relationships between donors and recipients [53]. However, some mothers remain skeptical about new feeding methods, preferring traditional practices and seeking additional evidence before accepting donor milk [54]. This hesitation is exacerbated when mothers perceive a lack of professional nursing guidance in the decision-making process.

In Muslim countries like Iran, the influence of authoritative figures—including both healthcare professionals and religious leaders—is paramount in shaping perspectives on HMB. Religious leaders play a critical role in addressing cultural and religious concerns that significantly influence maternal decision-making. Participants expressed anxieties about milk kinship, halal practices, and the donor’s lifestyle, including dietary habits, medication use, and ritual purity (ṭahārah), which are deeply rooted in Islamic ethical and religious frameworks. For example, concerns about milk kinship, as articulated by P1, highlight the fear of unintended social or religious consequences, such as future relationships between milk siblings. Endorsements from trusted religious scholars can help reassure families about the permissibility of HMB within Islamic guidelines, particularly by addressing issues such as halal compliance and the need for transparent documentation of milk kinship.

Similarly, healthcare professionals—including pediatricians and nurses—enhance trust by validating the safety of donor milk through transparent screening processes that address concerns about medication use, infectious diseases, and hygiene standards. Collaborative efforts between religious authorities and healthcare experts are essential to increase public confidence and cultural legitimacy of HMB. For instance, public awareness campaigns featuring *fatwas* or official statements from respected scholars, alongside endorsements by medical professionals, could legitimize HMB and alleviate maternal fears regarding both health risks and religious constraints. Such initiatives should emphasize the implementation of rigorous screening protocols, including assessments of medication use and adherence to halal dietary standards, to foster informed and culturally sensitive acceptance of milk banking in conservative Muslim societies.

Past behavioral patterns, such as prior reliance on formula feeding or informal milk-sharing networks, further shape mothers’ hesitancy toward HMB. Gribble [55] found that mothers with an insufficient milk supply often seek alternatives they perceive as superior to formula, underscoring how past experiences influence milk donation acceptance. These findings highlight the complex interplay between trust, prior experiences, and decision-making processes in determining the accessibility and utilization of HMB services.

The final theme, environmental factors, encompasses four subthemes: institutional accessibility, social support systems, authoritative influence, and cultural norms. These elements were identified as key barriers to HMB accessibility for nursing mothers. In Iran, HMB facilities are scarce, with only a few locations, such as Tabriz, offering these services. As a result, mothers in other regions face substantial challenges in accessing donor milk. Current literature highlights the value of a nonprofit, integrated HMB model that ensures equitable access to an exclusive human milk diet while prioritizing best practices in maternal and newborn care [9].

Institutional accessibility and authoritative influence also shape HMB perceptions. In the U.S., HMB gains legitimacy through its perceived association with medical organizations and governmental advocacy, even without direct affiliations. For instance, national health initiatives such as the Healthy People 2020 Objectives and the Surgeon General’s Call to Action to Support Breastfeeding [56] have driven demand for banked human milk. These efforts illustrate how authoritative influence can enhance HMB accessibility by framing it as a medically endorsed and socially supported practice.

The last subtheme, cultural norms, also emerged as a critical barrier to HMB accessibility. A recent study on Ghanaian immigrant women in the U.S. highlights how cultural beliefs shape attitudes toward donor milk [57]. Cultural norms can either facilitate or hinder HMB acceptance, depending on the societal context. In Iran, where HMB services are geographically limited, cultural attitudes may exacerbate disparities in access. Social support systems and community perceptions significantly influence mothers’ willingness to engage with HMB, as family and societal expectations often dictate individual behaviors. These findings underscore the importance of culturally sensitive interventions to enhance HMB accessibility, ensuring that institutional, social, and cultural barriers are effectively addressed to support nursing mothers.

### Implication

**Fig. 3:**
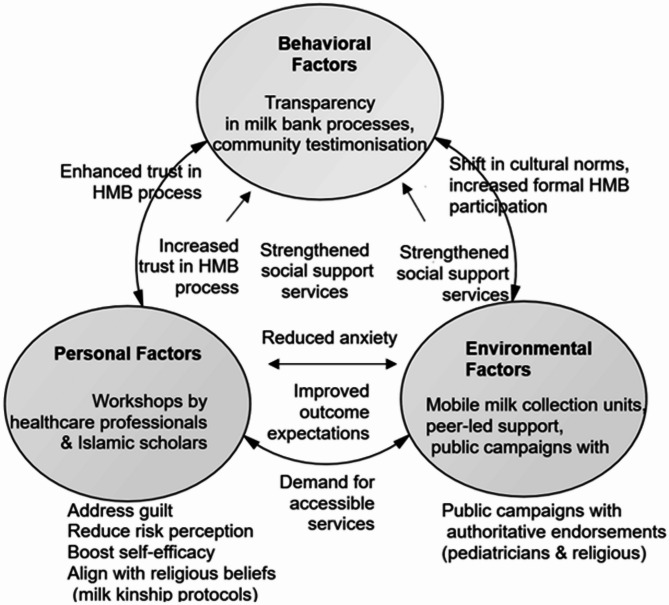
Culturally sensitive HMB empowerment program

The findings of this study, coupled with the “Culturally Sensitive HMB Empowerment Program” intervention illustrated in the Fig. 3, offer actionable strategies to enhance HMB acceptability among nursing mothers in Iran. For personal factors, implementing workshops led by healthcare professionals and Islamic scholars can directly address emotional barriers such as guilt and anxiety, while reducing risk perception through education on milk bank safety protocols. These workshops should also incorporate Islamic-compliant practices, such as milk kinship protocols, to align with religious beliefs, thereby fostering trust and improving self-efficacy among mothers. This approach not only empowers mothers to feel confident in their decision to engage with HMB but also creates a demand for accessible services, as mothers become more willing to participate when their personal concerns are addressed in a culturally sensitive manner.

On the environmental and behavioral fronts, the intervention highlights the need for systemic changes to improve HMB uptake. Deploying mobile milk collection units and integrating HMB services into local clinics can enhance institutional accessibility, making it easier for mothers to donate or receive milk without logistical burdens. Establishing peer-led support groups can strengthen social support systems, normalize HMB within communities, and shift cultural norms, while public campaigns featuring endorsements from paediatricians and religious leaders can leverage authoritative influence to build trust. By ensuring transparency in milk bank processes, such as demonstrating pasteurization and donor screening, healthcare providers can address trust-based decision-making, encouraging mothers to transition from informal milk-sharing to formal HMB participation. Collectively, these practical strategies create a supportive ecosystem where personal empowerment, environmental accessibility, and behavioral trust reciprocally reinforce each other, aligning with SCT’s triadic model to promote sustainable HMB adoption in Iran.

### Limitations and future direction

This qualitative study on the barriers to HMB acceptability among twelve nursing mothers of premature infants in Iran offers valuable insights into the cultural and religious context of HMB. However, the small sample size, drawn from two hospitals through purposive and snowball sampling, may not represent the broader diversity of Iranian nursing mothers, particularly those from rural areas or with varying levels of religious adherence. This limits the generalizability of the findings and may overlook regional or socioeconomic factors that influence HMB perceptions. Additionally, the focus on mothers of premature infants who could not breastfeed reduces the applicability of the findings to mothers of healthy or full-term infants. The semi-structured interview format may also be influenced by social desirability bias, as participants might have tailored their responses to align with cultural expectations. Moreover, the study did not explore the perspectives of other key stakeholders, such as religious scholars, healthcare providers, or family members, whose influence on maternal decision-making was evident. Future research should expand the sample size and include diverse regions to improve generalizability. A mixed-methods approach would provide a more comprehensive understanding of the factors influencing HMB acceptability, while exploring Islamic-compliant HMB protocols and community-based education could further enhance HMB adoption in Iran and similar contexts.

## Conclusion

This study illuminates the complex barriers to HMB acceptability among nursing mothers in Iran, revealing the intricate interplay of personal, environmental, and behavioral factors through the lens of SCT. By identifying key challenges—such as emotional guilt, religious concerns like milk kinship, limited institutional accessibility, and mistrust rooted in past informal sharing practices—the research underscores the need for culturally sensitive interventions tailored to Muslim-majority contexts. The proposed “Culturally Sensitive HMB Empowerment Program” offers a promising pathway by integrating Islamic-compliant protocols, community education, and enhanced service accessibility to foster trust and participation. These findings not only validate SCT’s triadic reciprocal causation model in a non-Western setting but also contribute to global efforts to improve neonatal health outcomes by bridging medical benefits with sociocultural realities. Ultimately, this study highlights the importance of addressing deeply rooted cultural and religious values to promote HMB as a viable solution for vulnerable infants in Iran, paving the way for more inclusive and effective maternal and child health initiatives.

## Data Availability

The datasets generated and/or analyzed during the current study are available from the corresponding author upon reasonable request.
